# Impact of previous COVID-19 infection on postoperative complications and functional recovery: a 1-year follow-up ambispective cohort study

**DOI:** 10.1097/JS9.0000000000001869

**Published:** 2024-06-27

**Authors:** Lu Che, Jiawen Yu, Di Jin, Xue Bai, Yi Wang, Yuelun Zhang, Li Xu, Le Shen, Yuguang Huang

**Affiliations:** a Department of Anesthesiology, Peking Union Medical College Hospital; b Department of Medical Records, Peking Union Medical College Hospital; c Medical Research Center, Peking Union Medical College Hospital, Beijing, China

**Keywords:** complications, COVID-19 infection, functional disability, postoperative recovery

## Abstract

**Background:**

It’s necessary to reassess the patients’ short-term and long-term postoperative outcomes in the post-COVID-19 era. This study aims to provide more comprehensive evidence regarding the timing of surgery after COVID-19 infection among the vaccinated population upon Omicron variant, considering the duration after acute COVID-19 infection, the severity of COVID-19, patients’ comorbidities, and the full course quality of postoperative recovery.

**Materials and methods:**

This is a single-center cohort study. Patients diagnosed with preoperative COVID-19 infection were consecutively included before surgery. Patients’ demographics, surgical characteristics, and COVID-19-related factors were documented. Exposure was the time interval from COVID-19 infection to surgery. The primary outcome was postoperative complications within 30 days. The secondary outcomes included postoperative mortality within 30 days, functional disability at 6 and 12 months after surgery.

**Results:**

A total of 4953 patients were included, and postoperative complications occurred in 353 patients (7.1%) within 30 days after surgery. Time interval from COVID-19 infection to surgery was associated with postoperative complications within 30 days after surgery [adjusted odds ratio (aOR) per day: 0.99; 95% CI, 0.99–1.00; *P*<0.01], as well as postoperative 6- and 12-month functional disability [(aOR): 1.00; 95% CI, 0.99–1.00; *P*<0.01; and (aOR) 0.99; 95% CI, 0.98–1.00; *P*=0.01, respectively). Delaying surgery beyond a time interval of 2 weeks was associated with reduced postoperative 30-day complications [(aOR): 0.63; 95% CI, 0.43–0.91; *P*=0.01] and mortality [(aOR): 0.07; 95% CI, 0.01–0.38; *P*<0.01]. Meanwhile, delaying surgery beyond a time interval of 7 weeks was associated with reduced functional disability at both 6-month [(aOR): 0.67; 95% CI, 0.58–0.79; *P*<0.01] and 12-month postoperatively [(aOR): 0.71; 95% CI, 0.53–0.95; *P*=0.02].

**Conclusion:**

A 2 weeks delay after COVID-19 infection is necessary for decreasing short-term postoperative risks, and a longer waiting period could be beneficial for long-term functional recovery.

## Introduction

Highlights

- Perioperative practitioners need to update the traditional understanding of postoperative recovery in this post-COVID-19 era and to optimize both short-term and long-term recovery.
- Post-COVID-19 infection interval in relation to surgery impact both early postoperative complication rate and long-term functional recovery.
- A time interval of 2 weeks after COVID-19 infection could provide a much safer surgical condition with a significant decrease of postoperative morbidities and mortalities within 30 days, while a time interval of 7 weeks after COVID-19 infection could be a better choice that leads to better long-term functional recovery.

Since its emergence in December 2019, SARS-CoV-2, which causes COVID-19, has had a significant impact on healthcare services worldwide^1–3^. Research collecting data from pre-vaccine, early phase of the COVID-19 pandemic indicated that postoperative morbidity and mortality increased significantly with perioperative COVID-19 infection^4,5^. Evidence from the COVIDSurg Collaborative suggested 30‐day mortality close to 20% and pulmonary complications morbidity around 50% in patients undergoing elective surgery with perioperative COVID-19 infection^4^. Consensus therefore recommended at least 7–8 weeks of delay for elective surgery among patients diagnosed with preoperative SARS-CoV-2 infection^6–8^.

Compared with prior SARS-CoV-2 variants in 2020, the circulating variant Omicron in 2022 revealed a greater rate of transmissibility^9^ and less severe clinical symptoms during acute infection^10^. Recognizing the adverse impact of unnecessary delays in surgery^11^ especially among cancer patients^12^, an updated issue in 2023^13^ suggested a shorter preoperative delay of 2 weeks after COVID-19 infection for low-risk elective surgeries. A delay of 2–7 weeks for patients receiving moderate-risk elective surgeries was appropriate, and for patients receiving high-risk elective surgeries or having ongoing COVID-19 symptoms a necessary delay should be 7 weeks or more. However, population-based evidence regarding postoperative morbidity and mortality after Omicron infection in the era of vaccination has not been thoroughly evaluated. Furthermore, no evidence regarding the long-term consequence of preoperative COVID-19 infection is currently available.

Existing data often lack comprehensive information on the severity and persistence of COVID-19 symptoms immediately preceding surgery, as well as vaccination status^1,14^. Moreover, outcome assessments in previous studies have predominantly focused on short-term mortality and respiratory outcomes^4,15^, which may not adequately reflect a patient’s perception of long-term recovery, and potentially overlooking other complications as well as crucial aspects of long-term consequences^16^.

With global surgical activity restored to normal, it is imperative to understand the impact of currently circulating SARS-CoV-2 variants on both short-term and long-term postoperative outcomes^17^. To address this gap, we conducted a single-center, cohort study to examine the correlation between the time interval from COVID-19 infection to surgery and the postoperative short-term outcome and long-term functional recovery.

## Materials and methods

### Study design

The study protocol was approved by the Institutional Review Board and registered at clinicaltrials.gov. The requirement for informed consent was waived by the Institutional Review Board. This was an ambispective cohort study with a published protocol^18^. The current study complies with the Declaration of Helsinki and has been reported in line with the STROCSS criteria^19^. Supplemental Digital Content 1, http://links.lww.com/JS9/C902.

### Participants

All patients scheduled to undergo surgery with anesthesia care at our hospital between 1 December 2022, and 27 February 2023, and diagnosed with COVID-19 infection before surgery were eligible for inclusion. This study period was chosen for the convenience of recruitment for a large number of surgical patients were diagnosed preoperative COVID-19 infection^20^, and most of them had confirmed SARS-CoV-2 test. Exclusion criteria were patients confirmed SARS-CoV-2 infection on the day of surgery or after, patients receiving ambulatory surgery or outpatient surgery, and patients unwilling to participate or provide COVID-19-related information. If multiple procedures were performed during the study period, the procedure performed nearest to the date of the first positive test was defined as the index procedure.

### Exposures and confounders

The primary exposure variable was the time interval from COVID-19 infection to surgery. COVID-19 infection was confirmed by one or more of the following methods: (a) positive SARS-CoV-2 reverse transcription-polymerase chain reaction (qRT-PCR) nasopharyngeal swab, (b) positive SARS-CoV-2 antigen test, or (c) clinical diagnosis made before surgery.

We documented factors associated with COVID-19 that have been identified as potential risk factors of surgical outcome^21^, including (1) Severity of infection categorized as mild (non-symptom-to-mild), moderate (requiring oxygenation), or severe (requiring ventilation support and/or hospitalization)^22^. (2) Vaccination status defined as receiving at least one vaccine dose before 14 days of the index operation date. (3) Presence of ongoing symptoms immediately before surgery. Other information collected included patient demographics and procedure-related factors, including age, sex, American Society of Anesthesiologists (ASA) physical status classification, comorbidities, type of surgery (major vs minor with definition provided in eTable 1, Supplemental Digital Content 2, http://links.lww.com/JS9/C903), surgical urgency (elective/emergency), and duration of anesthesia. Because we did not require preoperative pulmonary function tests for all participants and most of them received the surgery with normal cardiac function assessments, neither the direct impact of preoperative COVID-19 infection on preoperative lung function nor the New York Heart Association (NYHA) cardiac function was measured. To assess the impact of COVID-19 on the respiratory system and its implications for postoperative outcomes, we included ARISCAT (Assess Respiratory Risk in Surgical Patients in Catalonia) score categorizations. Patients with fewer than 26 points were classified as low risk, those with 26–44 points as intermediate risk, and those with scores of 45 points or higher as high risk^23^.

### Outcomes

The primary outcome was composite postoperative complications within 30 days of surgery. Included type of complication and definition can be found in supplemental material (eTable 2, Supplemental Digital Content 2, http://links.lww.com/JS9/C903). For better clinical relevance, the composite primary outcomes were further subcategorized into pulmonary complications (pneumonia, acute respiratory distress syndrome or acute respiratory failure, reintubation, unplanned use or prolongation of postoperative mechanical ventilation), cardiovascular complications (deep vein thrombosis, pulmonary embolism, myocardial infarction, new-onset arrhythmia, ischemic stroke, and acute kidney injury), and septic complications other than pulmonary infection (urinary tract infection, surgical site infection, and sepsis). Complication diagnoses were independently confirmed by at least two investigators, including a junior anesthesiologist (J.Y.), and a senior anesthesiologist (L.C.) and differences were adjudicated by a third senior anesthesiologist (L.S.).

Secondary outcomes included: (1) Postoperative 30-day mortality. (2) Functional disability evaluated at 6 months postoperatively. Functional disability was defined as the composite of mortality or a WHODAS 2.0 score equal to or exceeding 16%. The WHODAS 2.0 instrument assesses six domains through a total of 12 items, each scored on a five-point scale. The total 12-item score, transformed into a percentage of the total possible score, ranged from 0% (indicating no disability) to 100% (indicating maximal disability or death)^24,25^. A cut-off of 16% was validated within the surgical population^26–29^. (3) Functional disability assessed at 12 months postoperatively.

Patients were followed up via telephone 30 days, 6 months, and one year after surgery. At 30 days after surgery, the patients were followed up for the composite postoperative complications and the mortality. At 6 months and one year after surgery, the patients were followed up for the functional disability as defined.

### Statistical analysis

We conducted a descriptive analysis examining patient demographics, surgical characteristics, and COVID-19 exposure details. To assess the impact of the time interval from COVID-19 infection to surgery, we segmented all patients into five groups according to the time intervals of 0–2 weeks, 2–4 weeks, 4–6 weeks, 6–8 weeks, and over 8 weeks. To assess the association between the exposure and the primary outcome, we initially conducted regression analysis of postoperative complications against the time interval as a continuous variable. The relationship between the time interval from COVID-19 infection to surgery and the outcomes of interest were analyzed using a logistic regression model with a restricted cubic spline to test for non-linear relationship. If non-linear relationship exists, then a segmented regression model was further built to explore the potential breakpoint of the estimated risk of the primary and secondary outcome and the time interval from COVID-19 infection to surgery.

Subsequently, the cohort was stratified based on the specific cut-off points that were selected upon: (1) the segmented regression model built to explore the potential breakpoint of the estimated risk of the primary outcome and the time interval from COVID-19 infection to surgery, and (2) recent publications advocating for shorter preoperative delays^15^.

A multivariable logistic regression model was employed to evaluate the odds of developing the primary outcome, adjusting for confounders deemed clinically relevant a priori. These confounders included age, sex, comorbidities (hypertension, diabetes mellitus, coronary artery disease, congestive heart failure, stroke/transient ischemic attack, chronic renal failure, smoking, asthma, chronic obstructive pulmonary disease (COPD), peripheral vascular disease), ASA classification, surgical type (elective vs. emergency), anesthesia method, surgical risk classification (major vs. minor), and ARISCAT score. Additionally, COVID-19-related factors previously documented to influence early postoperative outcomes were adjusted for, including COVID-19 severity during the acute phase, vaccination against COVID-19 before surgery, and presence of ongoing symptoms immediately before surgery.

We compared the secondary outcomes of the other exposure variables using a similar confounding adjustment strategy except for including postoperative complications as covariates during analysis of the secondary outcome. To rule out the possibility the postoperative complication is a mediator of exposure and long-term consequences, we further performed sensitivity analysis.

Statistical significance was set at two-sided *P* less than 0.05. Data were analyzed using R (version 4.0.3, R Foundation for Statistical Computing, Vienna, Austria, www.r-project.org).

### Power analysis

According to our center’s surgical volume of inpatient surgery, ~5000 elective surgeries can be completed in our three-month study period. Assuming an 80% infection rate in the cohort, 4000 preoperative COVID-19 patients are expected to be enrolled. The original power analysis was conducted based on the comparison of postoperative complications between patients infected within 1 week and those infected longer than 1 week before surgery. Assuming a postoperative complication risk of 10.8% among patients with preoperative COVID-19 infection, and roughly 33% patients were infected within 1 week before surgery, a sample size of 4000 patients could provide 83.3% statistical power to detect an RR larger than 1.3, given a two-sided alpha of 0.05.

Using similar power analysis rationale, this sample size could provide 80% power to detect significant odds ratio (OR) larger than 1.49 or less than 0.57 between time interval less than 2 weeks and longer than 2 weeks or provide 84.8% to detect significant OR less than 0.996 or larger than 1.004 per one-day increase in time interval as continuous exposure assuming a mean of 47.4 and SD of 34.1.

## Results

**Figure 1:**
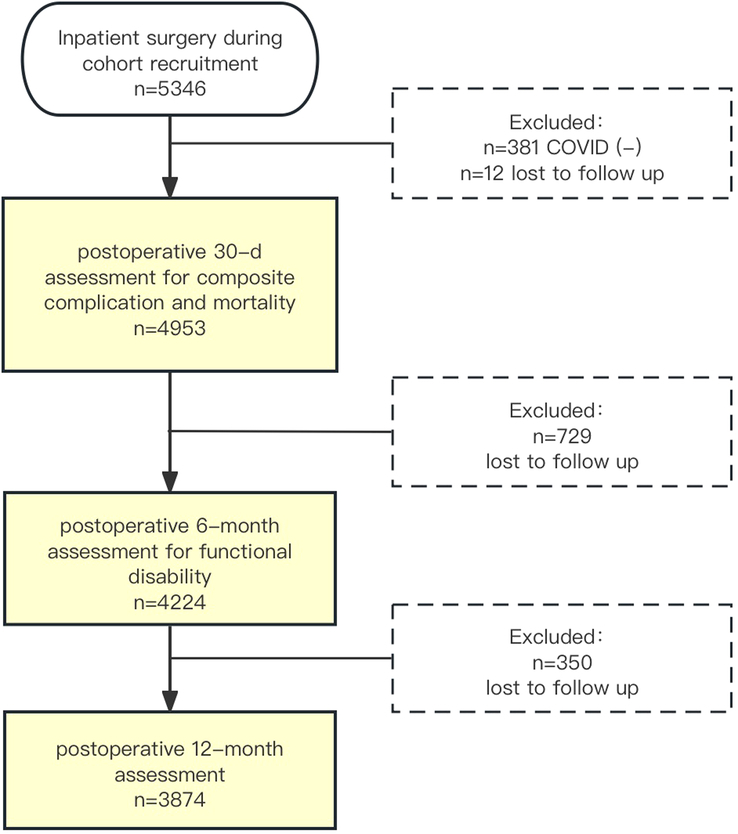
Flowchart of cohort creation.

During our study period, a total of 4953 patients were included in the analysis for primary outcome (Fig. 1). The median time interval from COVID-19 infection to surgery was 48 days (IQR, 26-62 days). Tables 1 and 2 present the baseline characteristics of the included patients and trends for each segmented time interval group. Overall, most patients (4805, 96.9%) were categorized as having mild COVID-19 symptoms (non-symptomatic to mild symptoms), while 75 (1.5%) were categorized as moderate (requiring oxygenation), and 14 (0.3%) as severe (requiring ventilation support and/or hospitalization). Of the 4953 patients included in the analysis, 4197 (84.7%) reported receiving at least one dose of COVID-19 vaccination at least 14 days before the index surgery. Most patients’ COVID-19 symptoms had resolved before surgery, while 521 patients (10.5%) still reported ongoing symptoms immediately before the procedure.

**Table 1:** Basic characteristics of the study population by the time interval quantiles between COVID-19^a^ diagnosis and surgery.

	Time interval between COVID-19 diagnosis and surgery					
	0–2weeks	2–4weeks	4–6weeks	6–8weeks	8weeks and over	Overall
Characteristics	*N*=421	*N*=1007	*N*=739	*N*=1010	*N*=1776	*N*=4953
Patient-related factor						
Mean (SD) age, years	46.40 (16.59)	46.00 (16.29)	45.00 (16.50)	46.63 (16.54)	46.28 (15.82)	46.11 (16.23)
Mean (SD) BMI, kg.m^−2^ ^a^	23.50 (4.10)	23.43 (4.02)	23.35 (3.67)	23.64 (3.75)	23.90 (3.94)	23.63 (3.90)
Sex, *n* (%)						
Male	130 (30.9)	264 (26.2)	191 (25.8)	299 (29.6)	549 (30.9)	1433 (28.9)
Female	291 (69.1)	743 (73.8)	548 (74.2)	711 (70.4)	1227 (69.1)	3520 (71.1)
Comorbidities, *n* (%)						
Active oncologic disease	127 (30.2)	327 (32.5)	212 (28.7)	313 (31.0)	528 (29.7)	1507 (30.4)
Hypertension	91 (21.6)	212 (21.1)	123 (16.6)	234 (23.2)	393 (22.1)	1053 (21.3)
Diabetes mellitus	61 (14.5)	106 (10.5)	66 (8.9)	109 (10.8)	176 (9.9)	518 (10.5)
Coronary artery disease	23 (5.5)	37 ( 3.7)	30 (4.1)	54 (5.3)	101 (5.7)	245 (4.9)
Congestive heart failure	2 (0.5)	7 (0.7)	4 (0.5)	5 (0.5)	6 (0.3)	24 (0.5)
Stroke/TIA	9 (2.1)	27 (2.7)	26 (3.5)	34 (3.4)	53 (3.0)	149 (3.0)
Renal failure	13 (3.1)	10 (1.0)	9 (1.2)	16 (1.6)	23 (1.3)	71 (1.4)
Smoker	36 (8.6)	51 (5.1)	28 (3.8)	68 (6.7)	104 (5.9)	287 (5.8)
Asthma	8 (1.9)	16 (1.6)	7 (0.9)	20 (2.0)	26 (1.5)	77 (1.6)
COPD	6 (1.4)	5 (0.5)	12 (1.6)	15 (1.5)	23 (1.3)	61 (1.2)
Peripheral vascular disease	6 (1.4)	16 (1.6)	21 (2.8)	27 (2.7)	56 (3.2)	126 (2.5)
Cognitive function impairment	2 (0.5)	5 (0.5)	3 (0.4)	3 (0.3)	7 (0.4)	20 (0.4)
ASA^b^ classification, *n* (%)						
I	77 (18.3)	220 (21.8)	176 (23.8)	231 (22.9)	398 (22.4)	1102 (22.2)
II	283 (67.2)	674 (66.9)	497 (67.3)	684 (67.7)	1228 (69.1)	3366 (68.0)
III	46 (10.9)	101 (10.0)	59 (8.0)	90 (8.9)	134 (7.5)	430 (8.7)
IV	13 (3.1)	12 (1.2)	7 (0.9)	5 (0.5)	16 (0.9)	53 (1.1)
V	2 (0.5)	0	0	0	0	2 (0.0)
ARISCAT scores, *n* (%)^c^						
High risk	12 (2.9)	21 (2.1)	13 (1.8)	22 (2.2)	26 (1.5)	94 (1.9)
Intermediate risk	70 (16.6)	161 (16.0)	111 (15.0)	160 (15.8)	260 (14.6)	762 (15.4)
Low risk	339 (80.5)	825 (81.9)	615 (83.2)	828 (82.0)	1490 (83.9)	4097 (82.7)
Procedure-related factor						
Case acuity scheduled (vs emergency), *n* (%)	355 (84.3)	936 (92.9)	669 (90.5)	957 (94.8)	1658 (93.4)	4575 (92.4)
Anesthetic method, *n* (%)						
General	372 (88.4)	933 (92.7)	680 (92.0)	950 (94.1)	1661 (93.5)	4596 (92.8)
Neuraxial	39 (9.3)	56 (5.6)	34 (4.6)	30 (3.0)	69 (3.9)	228 (4.6)
Monitored anesthesia care	8 (1.9)	17 (1.7)	25 (3.4)	26 (2.6)	43 (2.4)	119 (2.4)
Peripheral regional	2 (0.5)	1 (0.1)	0	4 (0.4)	3 (0.2)	10 (0.2)
Major surgery, *n* (%)	128 (30.4)	316 (31.4)	202 (27.3)	324 (32.1)	502 (28.3)	1472 (29.7)
Mean (SD) duration of surgery, minutes	99.54 (93.72)	102.57 (94.11)	97.21 (89.21)	103.84 (89.65)	98.15 (87.69)	100.19 (90.17)
Mean (SD) duration of anesthesia, minutes	148.75 (107.48)	150.40 (107.42)	143.47 (102.46)	150.73 (102.62)	144.70 (100.68)	147.25 (103.32)
COVID-19-related factor						
Vaccinated vs non-vaccinated	4197 (84.7)	331 (78.6)	858 (85.2)	635 (85.9)	853 (84.5)	1520 (85.6)
Covid-severity						
Mild	4804 (97.0)	405 (96.2)	981 (97.4)	708 (95.8)	976 (96.6)	1734 (97.6)
Moderate	75 (1.5)	6 (1.4)	13 (1.3)	16 (2.2)	18 (1.8)	22 (1.2)
Severe	74 (1.5)	10 (2.4)	13 (1.3)	15 (2.0)	16 (1.6)	20 (1.1)
Ongoing syndrome before surgery	521 (10.5)	91 (21.6)	136 (13.5)	92 (12.4)	79 (7.8)	123 (6.9)

COPD, chronic obstructive pulmonary disease; kg/m^−2^, kilogram per meter square; TIA, transient ischemic attack.

^a^ Body mass index (BMI) was calculated as the weight in kilograms divided by the square of the height in meters.

^b^ American society of Anesthesiologists (ASA)physical status ASA III: A patient with severe systemic disease; ASAII: A patient with severe systemic disease that is a constant threat to life; ASA III: A moribund patient who is not expected to survive without surgery; ASA IV: A patient with severe systemic disease that is a constant threat to life; ASA V: A moribund patient who is not expected to survive without surgery.

^c^ Assess Respiratory Risk in Surgical Patients in Catalonia (ARISCAT) scores were calculated based on age, preoperative SpO2, history of respiratory infection in the last month, preoperative anemia, surgical incision site, duration of surgery, and whether surgery was an emergency procedure.

**Table 2:** Incidence of primary and secondary outcome across preoperative infection interval group.

	Time interval between COVID-19 diagnosis and surgery, *n* (%)					
Characteristics	0–2weeks, *n* (%)	2–4weeks, *n* (%)	4–6weeks, *n* (%)	6–8weeks, *n* (%)	8weeks and over, *n* (%)	Overall, *n* (%)
Postoperative 30-day complication, *n*=4953	50 (11.9)	92 (9.1)	63 (8.5)	56 (5.5)	92 (5.2)	353 (7.1)
Cardiovascular complication	24 (5.7)	35 (3.5)	25 (3.4)	27 (2.7)	48 (2.7)	159 (3.2)
Respiratory complication	28 (6.7)	52 (5.2)	31 (4.2)	42 (4.2)	51 (2.9)	204 (4.1)
Infections complication	24 (5.7)	36 (3.6)	34 (4.6)	24 (2.4)	38 (2.1)	156 (3.1)
Postoperative 30-day mortality, *n*= 4953	8 (1.9)	4 (0.4)	0	1 (0.1)	0	13 (0.3)
Postoperative 6-month functional disability, *n*=4224	77 (24.4)	227 (27.3)	156 (25.4)	184 (21.1)	277 (17.8)	921 (22.0)
Postoperative 12-month functional disability, *n*= 3874	21 (6.9)	64 (8.0)	27 (4.8)	39 (4.9)	65 (4.6)	216 (5.6)

Within 30 days after surgery, the overall incidence of the postoperative complications was 7.1% (*n*=353) (Table 2). For the complication subcategory, pulmonary complications occurred in 204 (4.1%) patients. Cardiovascular complications occurred in 159 (3.2%) patients. 156 (3.1%) experienced septic complications. Thirteen patients (0.3%) died within 30 days after surgery. At the 6-month follow-up, 4224 patients completed assessments, yielding a response rate of 85.3%. Functional disability was reported in 21.8% of these cases (*n*=921). Subsequently, at the 12-month follow-up, 3874 patients completed assessments, resulting in a response rate of 78.2%. Among these, functional disabilities were reported in 5.6% of cases (*n*=216).

**Figure 2:**
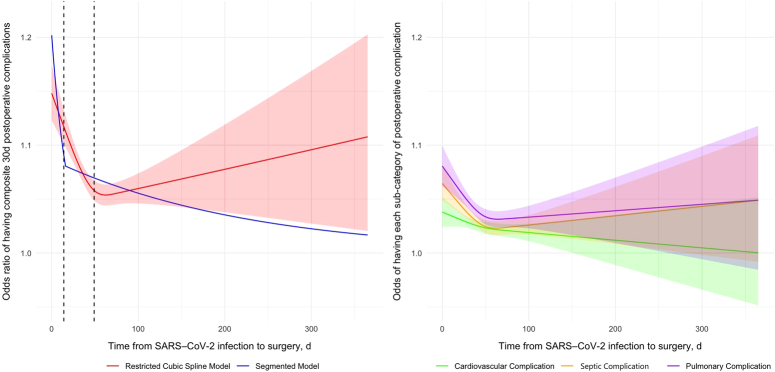
Odds ratio (OR) of experiencing primary outcome. Left panel: This panel displays the OR for 30-day postoperative complications, modeled using both a restricted cubic spline and a segmented approach. The red line indicates the OR predicted by the restricted cubic spline model with 3 knots for composite 30-day postoperative complications, with the shaded area representing the 95% CI. The blue line depicts the OR predicted by the segmented model. The vertical dashed lines denote the intervals of 2 weeks and 7 weeks between the diagnosis of SARS-CoV-2 infection and the surgical procedure. Right Panel: This panel presents the odds for each category of postoperative complications predicted by restricted cubic spline model with 3 knots. The green line with its shaded area indicates the OR and 95% CI for cardiovascular complications; the orange line and its shaded area indicates the OR and 95% CI for septic complications; and the purple line with its shaded area indicates the OR and 95% CI for respiratory complications.

**Figure 3:**
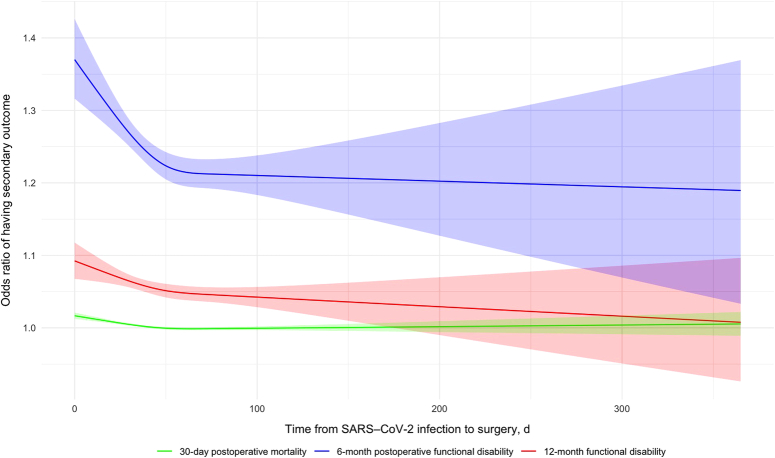
Odds ratio (OR) of having secondary outcome. This figure displays the predicted ORs for secondary outcomes post-surgery, using a restricted cubic spline model with 3 knots. The green line with its shaded area represents the OR and 95% CI for 30-day postoperative mortality. The blue line and its corresponding shaded area the OR and 95% CI for 6-month postoperative functional disability. Lastly, the red line with the shaded area illustrates the OR and 95% CI for 12-month postoperative functional disability, providing a comprehensive view of the risks associated with varying intervals between SARS-CoV-2 infection and surgical intervention.

The association between the time interval from COVID-19 infection to surgery and the odds of postoperative complications was non-linear using a restricted cubic spline model (Fig. 2). A potential breakpoint at 16.3 days (~2-week cut-off) was observed using a segmented regression model. Similarly, a non-linear association was revealed between the time interval from COVID-19 infection to surgery and the odds of postoperative 30-day mortality as well as postoperative functional disability at both 6 months and 12 months (Fig. 3). Segmented regression analysis identified breakpoints of 11 days, 79 days, and 46 days, respectively (eFigure 1-3, Supplemental Digital Content 2, http://links.lww.com/JS9/C903).

The time interval from COVID-19 infection to surgery exhibited a significant association with postoperative complications within 30 days [adjusted odds ratio (aOR) per day increase: 0.99; 95% CI, 0.99–1.00; *P*<0.01] (Table 3), after adjustment for confounding variables. Factors including patients’ age, comorbidities (renal failure, peripheral vascular disease and cognitive function impairment), ASA physical classification, surgery urgency, the duration of anesthesia, and ongoing symptoms all displayed significant correlation with the postoperative complications within 30 days.

**Table 3:** Full multivariate regression model of time interval between SARS-CoV-2 infection and surgery as continuous variable and postoperative complications.

Variable	aOR	95% CI	*P*
Time interval between COVID-19 diagnosis and surgery, days	0.99	0.99–1.00	0.01
Age, years	1.01	1.00–1.02	<0.01
Hypertension	0.99	0.72–1.36	0.96
Diabetes mellitus	1.25	0.89–1.77	0.2
Coronary artery disease	1.40	0.92–2.15	0.12
Congestive heart failure	1.39	0.47–4.11	0.56
Stroke/TIA	1.32	0.79–2.21	0.29
Renal failure	4.22	2.19–8.15	<0.01
Smoker	1.27	0.81–2.00	0.29
Asthma	0.18	0.02–1.37	0.1
COPD	0.77	0.33–1.80	0.54
Peripheral vascular disease	2.29	1.31–4.01	<0.01
Cognitive function impairment	4.90	1.38–17.4	0.01
ASA^a^ I	Reference		
ASA^a^ II	0.99	0.65–1.50	0.94
ASA III	2.17	1.28–3.69	<0.01
ASA IV	5.52	2.37–12.82	<0.01
ASA V	0.57	0.01–22.81	0.77
Male sex	0.92	0.7 0–1.23	0.59
Elective surgery	3.54	2.38~5.28	<0.01
General anesthesia	Reference		
Monitored anesthesia care	0.39	0.16–1.00	0.05
Neura-axial analgesia	0.77	0.41–1.45	0.41
Peripheral nerve block	0.32	0.03–3.22	0.33
ARISCAT^b^ low risk	Reference		
ARISCAT^b^ intermediate risk	1.32	0.70–2.49	0.4
ARISCAT high risk	0.92	0.47–1.80	0.8
BMI, kg.m^−2^	1.00	0.97–1.04	0.83
Anesthesia duration, min	1.00	1.00–1.01	<0.01
Major surgery	1.19	0.90–1.59	0.23
COVID-19 severity^c^	1.27	0.91–1.77	0.16
Vaccination	0.89	0.65–1.24	0.5
Ongoing symptom	1.48	1.04–2.12	0.03

aOR, adjusted odds ratio; COPD, chronic obstructive pulmonary disease; kg/m^−2^, kilogram per meter square; TIA, transient ischemic attack.

^a^ American society of Anesthesiologists (ASA) physical status ASA III: A patient with severe systemic disease; ASAII: A patient with severe systemic disease that is a constant threat to life; ASA III: A moribund patient who is not expected to survive without surgery; ASA IV: A patient with severe systemic disease that is a constant threat to life; ASA V: A moribund patient who is not expected to survive without surgery.

^b^ Assess Respiratory Risk in Surgical Patients in Catalonia (ARISCAT) scores were calculated based on age, preoperative SpO2, history of respiratory infection in the last month, preoperative anemia, surgical incision site, duration of surgery, and whether surgery was an emergency procedure.

^c^ Incorporated as continuous variable, mild=1, moderate=2,severe=3.

For the secondary outcomes, the time interval from COVID-19 infection to surgery also exhibited a significant association with postoperative mortality within 30 days [(aOR) per day increase 0.89:0.89; 95% CI, 0.80–1.00; *P*=0.04] (Table 4), postoperative functional disability at 6 months [(aOR) per day increase: 1.00; 95% CI, 0.99–1.00; *P*<0.01] (Table 5) and postoperative functional disability at 12 months [(aOR) per day increase: 0.99; 95% CI, 0.98–1.00; *P*=0.01] (Table 6) following surgery, after adjustment for confounding variables. Meanwhile, history of having postoperative 30-day complications displayed a significant correlation with functional disability at 6 months and 12 months after the surgery.

**Table 4:** Full multivariate regression model of time interval between SARS-CoV-2 infection and surgery and postoperative 30-day mortality^a^.

Variable	aOR	95% CI	*P*
Time interval between COVID-19 diagnosis and surgery, days	0.89	0.00–Inf	0.04
Age, years	0.95	0.80–0.10	0.40
Hypertension	16.97	0.85–1.07	0.08
Diabetes mellitus	0.67	0.71–407.05	0.74
Coronary artery disease	0.23	0.06–7.35	0.36
Congestive heart failure	0.01	0.01–5.47	0.09
Stroke/TIA	1.95	2.21×10^−5^–2.21	0.71
Renal failure	77.47	6.23×10^−2^–62.26	0.09
Smoker	0.02	0.51–11.79×10^3^	0.19
Asthma	0.00	3.01×10^−5^–7.82	1.00
COPD	320.38	0.00–Inf	0.09
Peripheral vascular disease	0.10	0.43–2.39×10^5^	0.35
Cognitive function impairment	148.16	6.93×10^-4^–13.59	0.11
ASA^b^ I	Reference		
ASA^b^ II	5.34×10^6^	0.34–6.51×10^4^	1.00
ASA III	2.39×10^7^	0.00–Inf	1.00
ASA IV	8.02×10^8^	00.00–Inf	1.00
ASA V	8.02×10^8^	0.00–Inf	0.99
Male sex	3.83×10^10^	0.00–Inf	0.30
Elective surgery	4.30	0.28–66.62	0.09
General anesthesia	Reference		
Monitored anesthesia care	0.01	1.76× 10^-5^–2.18	1.00
Neura-axial analgesia	NA		
Peripheral nerve block	0.00	0.00–Inf	1.00
ARISCAT^c^ low risk	Reference		
ARISCAT^c^ intermediate risk	2.35	0.00–Inf	0.56
ARISCAT high risk	0.00	0.13–41.73	0.99
BMI, kg.m^−2^	0.71	0.00–Inf	0.05
Anesthesia duration, min	1.00	0.50–0.99	0.20
Major surgery	37.01	0.99–1.01	0.04
COVID-19 severity ^d^	1.19	1.16–1.18× 10^3^	0.83
Vaccination	0.04	0.24–5.74	0.03
Ongoing symptom	1.62	2.68× 10^-3^–0.71	0.73

aOR, adjusted odds ratio; COPD, chronic obstructive pulmonary disease; kg/m^−2^, kilogram per meter square; TIA, Transient ischemic attack.

^a^ Because the very limited number of death events, estimated confidence intervals are wide and should be interpreted with causion.

^b^ American society of Anesthesiologists (ASA) physical status ASA III: A patient with severe systemic disease; ASAII: A patient with severe systemic disease that is a constant threat to life; ASA III: A moribund patient who is not expected to survive without surgery; ASA IV: A patient with severe systemic disease that is a constant threat to life; ASA V: A moribund patient who is not expected to survive without surgery.

^c^ Assess Respiratory Risk in Surgical Patients in Catalonia (ARISCAT) scores were calculated based on age, preoperative SpO2, history of respiratory infection in the last month, preoperative anemia, surgical incision site, duration of surgery, and whether surgery was an emergency procedure.

^d^ Incorporated as continuous variable, mild=1, moderate=2,severe=3.

**Table 5:** Full multivariate regression model of time interval between SARS-CoV-2 infection and surgery and postoperative 6-month functional disability.

Variable	aOR	95% CI	*P*
Time interval between COVID-19 diagnosis and surgery, days	1.00	0.99–1.00	<0.01
Age, years	1.03	1.02–1.04	<0.01
Hypertension	0.86	0.69–1.06	0.15
Diabetes mellitus	0.99	0.77–1.27	0.93
Coronary artery disease	1.02	0.72–1.44	0.93
Congestive heart failure	0.19	0.05–0.67	0.01
Stroke/TIA	1.53	1.02–2.29	0.04
Renal failure	1.67	0.90–3.10	0.10
Smoker	1.06	0.76–1.48	0.74
Asthma	1.67	0.94–2.95	0.08
COPD	0.94	0.49–1.78	0.84
Peripheral vascular disease	1.01	0.62–1.67	0.96
Cognitive function impairment	2.05	0.51–8.22	0.31
ASA^a^ I	Reference		
ASA^a^ II	1.25	0.98–1.58	0.07
ASA III	1.65	1.15–2.38	0.01
ASA IV	0.95	0.38–2.35	0.91
ASA V	NA		
Male sex	0.70	0.58–0.85	<0.01
Elective surgery	1.24	0.88–1.74	0.22
General anesthesia	Reference		
Monitored anesthesia care	1.12	0.67–1.87	0.66
Neura-axial analgesia	1.08	0.70–1.66	0.72
Peripheral nerve block	7.69	0.88–66.82	0.06
ARISCAT^b^ low risk	Reference		
ARISCAT^b^ intermediate risk	1.21	0.67–2.19	0.52
ARISCAT high risk	1.76	0.97–3.22	0.06
Body mass index, kg.m^−2^	0.98	0.96–1.01	0.16
Anesthesia duration, min	1.00	1.00–1.00	<0.01
Major surgery	1.49	1.24–1.79	<0.01
COVID-19 severity	1.81	1.36–2.41	<0.01
Vaccination	0.71	0.58–0.88	<0.01
Ongoing symptom	1.07	0.84–1.37	0.57
Having postoperative 30-day complication	1.85	1.39–2.47	<0.01

aOR, adjusted odds ratio; COPD, chronic obstructive pulmonary disease; kg/m^−2^, kilogram per meter square; TIA, transient ischemic attack.

^a^ American society of Anesthesiologists(ASA) physical status ASA III: A patient with severe systemic disease; ASAII: A patient with severe systemic disease that is a constant threat to life; ASA III: A moribund patient who is not expected to survive without surgery; ASA IV: A patient with severe systemic disease that is a constant threat to life; ASA V: A moribund patient who is not expected to survive without surgery.

^b^ Assess Respiratory Risk in Surgical Patients in Catalonia (ARISCAT) scores were calculated based on age, preoperative SpO2, history of respiratory infection in the last month, preoperative anemia, surgical incision site, duration of surgery, and whether surgery was an emergency procedure.

**Table 6:** Full multivariate regression model of time interval between SARS-CoV-2 infection and surgery and postoperative 12-month functional disability.

Variable	aOR	95% CI	*P*
Time interval between COVID-19 diagnosis and surgery, days	0.99	0.99–1.00	0.01
Age, years	1.05	1.04–1.07	<0.01
Hypertension	0.80	0.56–1.15	0.23
Diabetes mellitus	0.91	0.60–1.38	0.66
Coronary artery disease	0.82	0.48–1.4	0.47
Congestive heart failure	0.17	0.03–0.97	0.05
Stroke/TIA	2.32	1.35–3.99	<0.01
Renal failure	1.63	0.65–4.08	0.30
Smoker	1.40	0.80–2.43	0.23
Asthma	0.77	0.21–2.79	0.69
COPD	1.29	0.53–3.18	0.58
Peripheral vascular disease	1.68	0.85–3.32	0.14
Cognitive function impairment	1.43	0.13–15.68	0.77
ASA^a^ I	Reference		
ASA^a^ II	1.68	0.89–3.16	0.11
ASA III	2.52	1.18–5.38	0.02
ASA IV	2.85	0.76–10.73	0.12
Male sex	0.56	0.39–0.81	<0.01
Elective surgery	2.02	1.11–3.67	0.02
General anesthesia	Reference		
Monitored anesthesia care	1.32	0.61–2.82	0.48
Neura-axial analgesia	0.48	0.17–1.31	0.15
Peripheral nerve block	5.44	0.82–36.24	0.08
ARISCAT^b^ low risk	Reference		
ARISCAT intermediate risk	1.66	0.64–4.32	0.30
ARISCAT high risk	2.25	0.84–6.08	0.11
BMI, kg.m^−2^	0.98	0.94–1.02	0.37
Anesthesia duration, min	1.00	1.00–1.00	<0.01
Major surgery	1.13	0.81–1.59	0.47
COVID-19 severity	2.11	1.45–3.07	<0.01
Vaccination	0.63	0.43–0.91	0.01
Ongoing symptom	1.29	0.83–2.00	0.26
Having postoperative 30-day complication	1.51	0.95–2.38	0.08

aOR, adjusted odds ratio; COPD, chronic obstructive pulmonary disease; kg/m^−2^, kilogram per meter square; TIA, transient ischemic attack.

^a^ American society of Anesthesiologists (ASA) physical status ASA III: A patient with severe systemic disease; ASAII: A patient with severe systemic disease that is a constant threat to life; ASA III: A moribund patient who is not expected to survive without surgery; ASA IV: A patient with severe systemic disease that is a constant threat to life; ASA V: A moribund patient who is not expected to survive without surgery.

^b^ Assess Respiratory Risk in Surgical Patients in Catalonia (ARISCAT) scores were calculated based on age, preoperative SpO2, history of respiratory infection in the last month, preoperative anemia, surgical incision site, duration of surgery, and whether surgery was an emergency procedure.

When taking patients with the time interval from COVID-19 infection to surgery less than 2 weeks as the reference group, the time intervals longer than 2 weeks were associated with reduced risks of postoperative complications [(aOR), 0.63; 95% (CI), 0.43–0.91; *P*=0.01] and postoperative mortality [(aOR), 0.63; 95% (CI), 0.43–0.92; *P*=0.02] within 30 days after surgery (Table 7). When taking patients with the time interval from COVID-19 infection to surgery less than 7 weeks as the reference group, the time intervals longer than 7 weeks was also associated with reduced risks of postoperative complications within 30 days [(aOR), 0.56; 95% (CI), 0.44–0.72; *P*<0.01], as well as less functional disability at 6 months [(aOR), 0.67; 95% (CI), 0.58–0.79; *P*<0.01] and 12 months [(aOR), 0.71; 95% (CI), 0.53–0.95; *P*=0.02] after surgery (Table 7).

**Table 7:** Association between preoperative COVID-19 diagnosis interval and primary and secondary outcomes.

	COVID-19 diagnosis to surgery time interval					COVID-19 diagnosis to surgery time interval				
Variable	<2 weeks	≥2 weeks	Adjusted OR	95% CI	*P*	<7 weeks	≥7 weeks	Adjusted OR	95% CI	*P*
Primary outcome										
30-day postoperative composite complication, *n* (%)	50 (11.9)	303 (6.7)	0.66	0.46–0.97	0.03	234 (9.0)	119 (5.1)	0.58	0.45–0.75	<0.01
Complication subcategory										
30-day postoperative pulmonary complication, *n* (%)	28 (6.7)	176 (3.9)	0.79	0.46–1.33	0.37	132 (5.1)	72 (3.1)	0.68	0.48–0.95	0.02
30-day postoperative cardiovascular complication, *n* (%)	24 (5.7)	135 (3.0)	0.60	0.36–1.03	0.06	97 (3.7)	62 (2.6)	0.84	0.58–1.20	0.33
30-day postoperative non-pneumonia infectious complication, *n* (%)	24 (5.7)	132 (2.9)	0.65	0.40–1.06	0.08	109 (4.2)	47 (2.0)	0.52	0.37–0.75	<0.01
Secondary outcome										
30-day mortality, *n* (%)	8 (1.9)	5 (0.1)	0.10	0.01–0.64	0.02	12 (0.5)	1 (0.0)	0.16	0.01–2.12	0.16
6-month functional disability, *n* (%)	77 (24.4)	844 (21.8)	0.93	0.69–1.23	0.60	538 (25.2)	383 (18.7)	0.69	0.59–0.81	<0.01
12-month functional disability, *n* (%)	21 (6.9)	195 (5.5)	0.83	0.50–1.38	0.48	131 (6.5)	85 (4.6)	0.73	0.54–0.99	0.04

Adjusted for age, sex, comorbidity of hypertension, diabetes mellitus, coronary artery disease, congestive heart failure stroke/TIA, chronic renal failure, smoking, asthma, COPD, peripheral vascular disease, ARISCAT score, ASA classification, type of surgery (surgery vs. emergency), anesthesia method, and surgical risk classification(major vs minor), vaccination against COVID-19 before surgery, and presence of ongoing symptoms immediately before surgery.

ASA, American society of Anesthesiologists; COPD, chronic obstructive pulmonary disease; OR, odds ratio; TIA, transient ischemic attack.

### Sensitivity analysis

To assess whether the inclusion of patients with a clinical diagnosis with COVID-19 would affect our results, we performed further sensitivity analysis. Among the included patients, 3840 (77.5%) were preoperatively diagnosed with a laboratory-confirmed COVID-19 infection using either a positive qRT-PCR nasopharyngeal swab (*n*=3172) or a positive antigen test (*n*=668). The association between the time interval and postoperative 30-day complications persisted among patients with laboratory-confirmed results [(aOR) of 0.48; 95% (CI) of 0.36–0.63; *P*<0.01]. Sensitivity analysis was also conducted to assess the inclusion of postoperative 30-day complications as a confounder for postoperative 6- and 12-month functional disability. The analysis showed that including postoperative 30-day complications as a confounder did not alter the results regarding the association with long-term functional recovery (eTable 3, Supplemental Digital Content 2, http://links.lww.com/JS9/C903)

## Discussion

Due to widespread vaccination among the population, less virulent circulating variants, and an increased call for access to safe surgery, up-to-date evidence on the impact of preoperative SARS-CoV-2 infection on postoperative early and late outcomes is needed.

The current cohort provides a more granular context compared to previous evidence generated from international surveys or administrative databases by documenting a wide array of patient-related, procedure-related as well as COVID-19-related factors while examining not only the early phase postoperative risk but also the long-term recovery risks.

Notably, we observed a non-linear association between the time interval from COVID-19 infection to surgery and postoperative complications, together with less overall postoperative morbidities and mortality compared to studies performed in the pre-Omicron and pre-vaccination era^4,5^. This finding aligns with our expectations and is consistent with previous research^1,17^.

In our cohort, the overall early postoperative risks significantly decreased after a 2-week delay, providing evidence supporting a multidisciplinary consensus statement issued in 2023^30^. Through observation of incidents of subcategories of complications across different time interval groups, cardiovascular, respiratory, and septic complications all demonstrated a decreasing trend with longer time interval from COVID-19 infection to surgery. The risk of cardiovascular complications seems to decrease after a 2-week delay, while the risk of pulmonary and septic complications began to show a trend of decrease after more than 4 weeks of delay. Pulmonary sequelae after COVID-19 infection may persist longer than other organ/system complications. Evidence suggested that altered diffusion capacity, restrictive pattern, and obstructive pattern were found in 39%, 15%, and 7%, respectively, of post-COVID-19 infection patients even after discharge from initial hospitalization^31^. This finding is similar to another study demonstrating that postoperative pulmonary complications remain elevated in patients even after delaying surgery for 5 or more weeks after a positive SARS-CoV-2 test^32^.

A recently published multicentre cohort study conducted from 2020 to 2021 in the United States, utilizing propensity score matching to contrast patients with and without preoperative COVID-19 infection, concluded risk of pulmonary complication and mortality should not be a concern after 2 weeks delay^15^. By employing a segmented model in our cohort conducted from 2022 to 2023, the breakpoint was ascertained upon the risk of postoperative complications significantly shifts. We further confirmed 2 weeks delay as a reasonable and safe cut-off value.

This study additionally revealed that a longer preoperative delay beyond 7 weeks might be more beneficial for not only mitigating immediate postoperative risks but also improving long-term outcomes and enhanced functional recovery. The long-lasting effects of COVID-19 infection on non-surgical populations have been extensively documented and studied^33,34^. The immediate post-COVID-19 multisystemic organ damage^4,5,17^, which can persist long after patient recovery, may be exacerbated by the inflammatory response induced by surgical stress, leading to additional risks in long-term recovery^35^.

When facilitating individualized risk assessment, factors correlated with the severity and prognosis of COVID-19 infection, such as older age and comorbidities might influence the postoperative outcome too. However, the severity of COVID-19 infection itself showed an aOR of 1.27 with a 95% CI of 0.91–1.77 and a *P* value of 0.16 (Table 3). This indicates that although the severity of preoperative COVID-19 infection seems to be associated with postoperative complications within 30 days after surgery, the association does not achieve statistical significance after adjusting for potential confounders, possibly due to the limited statistical power.

Besides, others also suggested that higher ASA classification, longer surgery or anesthesia time and having ongoing symptoms immediately before surgery could also increase the risk of early postoperative complications^6,36^. These factors should be considered regarding the optimal timing of surgery for patients with previous COVID-19 infection. However, it is important to note that while longer preoperative delays may lead to better short-term and long-term outcomes, they may not outweigh the benefits of early surgery in time-sensitive cases^37,38^

This study has some strengths. Determining the impact of COVID-19 infection on postoperative outcomes is challenging and requires a large cohort representing different surgical procedures. This study performed rapid recruitment of all inpatient surgical patients in three months, ensuring the inclusion of individuals with consistent laboratory-confirmed SARS-CoV-2 Omicron variant tests and reducing ascertainment bias. We produced a large sample size, primarily comprising patients infected with COVID-19 within 2 months before surgery, enhancing statistical power and generalizability. The inclusion of a diverse surgical population primarily affected by the SARS-CoV-2 Omicron variant^20,39^ reflects the current epidemiological landscape and high vaccination rates. Lastly, the use of a composite outcome allowed a comprehensive assessment of perioperative risk, facilitating informed perioperative planning.

This study also had limitations. This is a single-center study therefore limiting the generalizability of findings to other settings and populations. The absence of genome sequencing to identify SARS-CoV-2 lineages may limit specificity in variant identification. Furthermore, the inclusion of COVID-19 infection based on clinical symptoms may introduce false positives. Sensitivity analyses were performed to minimize this potential bias. The statistical significance of multiple secondary outcomes should be regarded with caution because they were not adjusted for multiplicity.

## Conclusion

In summary, we recommend that elective surgery be postponed to 2 weeks after COVID-19 infection. For patients with known risk factors associated with COVID-19 infection or elective surgery aiming for better long-term function recovery, 7 weeks of delay are suggested. We believe this study will provide crucial insights to inform future preoperative risk assessments for the Omicron variant of SARS-CoV-2 infection within the immunized surgical population.

## Ethical approval

The study protocol was approved by the Institutional Review Board of the Peking Union Medical College Hospital (IRB K3570) and registered at clinicaltrials.gov (NCT05689840).

## Consent

The requirement for informed consent was waived by the Institutional Review Board.

## Source of funding

This research was funded by the National High-Level Hospital Clinical Research Funding of Peking Union Medical College Hospital [2022-PUMCH-B-007]. The funders had no role in the study design, data collection, data analysis, data interpretation, or writing of the report. The lead author affirms that the manuscript is honest and accurate.

## Author contribution

L.C. helped with data curation, formal analysis, investigation, methodology, software, validation, writing—original draft and writing—review and editing. J.Y. helped with data curation, writing—original draft and writing—review and editing. D.J. helped with data curation, writing—original draft. X.B. helped with data curation and writing—review and editing. Y.W. helped with data curation and writing—review and editing. Y.Z. helped with data curation, formal analysis, methodology, software and writing—review and editing. L.X. helped with conceptualization and writing—review and editing. L.S. helped with conceptualization, funding acquisition, supervision, writing—original draft and writing—review and editing. Y.H. helped with conceptualization and writing—review and editing.

## Conflicts of interest disclosure

The authors declare no conflicts of interest.

## Research registration unique identifying number (UIN)

This study was registered at https://www.clinicaltrials.gov/clinicaltrials.gov (NCT05689840).

## Guarantor

Le Shen.

## Data availability statement

Research data supporting this publication are available upon request from the corresponding author(pumchshenle@163.com).

## Provenance and peer review

The paper was not commissioned, externally peer-reviewed.

## Supplementary Material
