# Supplementary material for: Impact of previous COVID-19 infection on postoperative complications and functional recovery: a 1-year follow-up ambispective cohort study
Source: Int J Surg. 2024 Jun 27;111(1):481–91. doi: 10.1097/JS9.0000000000001869 (PMC11745778; doi:10.1097/JS9.0000000000001869)
Supplement: Supplementary file 2 [file js9-111-0481-s002.pdf]

SUPPLEMENTARY MATERIALS

Impact of previous COVID-19 infection on postoperative complications and functional recovery: a one-year follow-up ambispective study

eTable1 Definitions of major surgeries

eTable2 Definitions of primary outcomes

eTable3 Sensitivity analysis for incorporating post-operative 30-day compilation as a confounding factor for long-term functional disability.

eFigure1 Odds of having 6-month postoperative functional disability

eFigure1 Odds of having 6-month postoperative functional disability

eFigure2 Odds of having 12-month postoperative functional disability

SUPPLEMENTARY TABLES

eTable1 Definitions of major surgery.

**Major surgery**	Major surgeries include lung resection (lobectomy, pneumonectomy, and segmentectomy), esophagectomy, mastectomy, colorectal resection for cancer (colectomy and proctectomy), prostatectomy, pancreatic resections (Whipple, total pancreatectomy, and distal pancreatectomies), hepatectomy, gastrectomy, hip replacement, coronary artery bypass grafting, knee replacement, laminectomy, hysterectomy, spinal fusion, elective open repair of abdominal aortic aneurysm (AAA), elective endovascular repair of abdominal aortic aneurysm (EVAR), brain tumor resection, and carotid endarterectomy.

The detailed description of each complication is listed as follows.

eTable2 Definitions of primary outcomes.

Pneumonia	1. At least one of the following symptoms:<br>a. Fever > 38°C with no other cause<br>b. Leukopenia (< 4000 WBC/mm^3) or leucocytosis (≥ 12 000 WBC/mm3)<br>c. And at least one of the following (if microbiological confirmation) or two of the following (if clinical definition only) symptoms:<br>i. new onset of purulent sputum, or change in character of sputum (colour, odour, quantity, consistency)<br>ii. cough or dyspnoea or tachypnoea.<br>iii. suggestive auscultation (rales or bronchial breath sounds), rhonchi, wheezing<br>iv. worsening gas exchange (e.g. O2 desaturation or increased oxygen requirements or increased ventilation demand).<br>2. AND:<br>a. One chest X-ray or CT-scan with a suggestive image of pneumonia for patients without underlying cardiac or pulmonary disease.<br>b. Two or more serial chest X-rays or CT-scans with a suggestive image of pneumonia for patients with underlying cardiac or pulmonary disease.<br>3. AND one of the following microbiological criteria:<br>a. No positive microbiological sample or qualitative positive sputum culture or lower respiratory tract specimen culture ("clinically defined healthcare associated pneumonia")<br>b. Bacteriologically confirmed diagnostic, by:<br>i. Positive quantitative culture from minimally contaminated lower respiratory tract specimen:<br>1. Broncho-alveolar lavage (BAL) with a threshold of ≥ 10^4 colony forming units

	(CFU)/ml or ≥ 5% of BAL-obtained cells contain intracellular bacteria on direct microscopic exam<br>2. protected brush with a threshold of ≥ 10^3 CFU/ml<br>3. distal protected aspirate with a threshold of ≥ 10^3 CFU/ml.<br>ii. Positive quantitative culture from possibly contaminated lower respiratory tract specimen:<br>1. Quantitative culture of endotracheal aspirate with a threshold of 10^6 CFU/ml.<br>iii. Alternative microbiology methods:<br>1. Positive blood culture not related to another source of infection.<br>2. Positive growth in culture of pleural fluid<br>3. Pleural or pulmonary abscess with positive needle aspiration<br>4. Histologic pulmonary exam shows evidence of pneumonia.<br>c. Positive exams for pneumonia with virus or particular germs: positive detection of viral antigen or antibody from respiratory secretions; positive direct exam or positive culture from bronchial secretions or tissue; seroconversion; detection of antigens in urine.<br>4. AND definitive administration of an antimicrobial treatment (i.e. confirmed therapy) decided by the attending physician for at least 72 hours.
Acute Respiratory Failure	Acute impairment in gas exchange between the lungs and the blood causing hypoxia with or without hypercapnia, defined as follows:<br>1. hypoxia with an arterial partial pressure of oxygen (PaO_2) of <60 mmHg) on room air or room air oxygen saturation less than or equal to 90%, or a PaO2 / FiO2 ratio < 300 mmHg.<br>2. AND at least one of the following symptoms:

	a. shortness of breath, tachypnoea, cyanosis, coughing or wheezing<br>b. anxiety, confusion, restlessness or drowsiness, severe headache<br>c. tachycardia, cardiac dysfunction, arrhythmia.
Unplanned reintubation or prolongation of postoperative mechanical ventilation	Any unplanned reintubation after surgery or prolonged mechanical ventilation its duration (for instance requirement of invasive mechanical ventilation beyond the usual first 12 postoperative hours which is our facility routine etc.).
Myocardial infarction	MI was defined as an absolute increase from a preoperative value (or between two postoperative measurements if the preoperative measurement was missing) of ≥ 14 ng/L for hs-cTnT and ≥ 45 ng/L for s-cTnI (the respective 99th percentile of both assays)
Newly onset arrhythmia	1. Narrow and broad complex tachy- and bradyarrhythmias that cause cause hemodynamic instability.<br>**2.** Atrial fibrillation that is not detected preoperatively.
Ischemic stroke/TIA	Brain infarction of ischemic etiology, or transient ischemic attack (TIA) which occurs during surgery or within 30 days after surgery, including the development of stroke after recovering from anesthesia that is diagnosed by a neurology specialist.
Deep vein thrombosis	Acute embolism and thrombosis of unspecified deep veins of unspecified lower extremity that is diagnosed by a specialist;
Pulmonary embolism	Pulmonary embolism not known preoperatively, whose diagnosis:<br>1. is suspected on usual clinical signs (non-exhaustive list): shortness of breath, dyspnoea, chest pain, cough, haemoptysis, leg pain or oedema, tachycardia, excessive sweating, lightheadedness, dizziness or passing out, cyanosis, etc.

	2. possibly reinforced by first line paraclinical examinations such as arterial blood gases, D-Dimer dosage, electrocardiogram, leg doppler ultrasound (showing deep vein thrombosis), etc.<br>3. AND definitely confirmed using at least one of the following diagnostic tests:<br>a. Computed tomographic pulmonary angiography.<br>b. Pulmonary V/Q lung scanning<br>c. Echocardiography visualizing embolized thrombi in right heart chambers or central pulmonary arteries<br>d. Pulmonary angiography.
Acute kidney injury	By the KDIGO definition, AKI is diagnosed by an absolute increase in sCr, at least 0.3 mg/dL (26.5 μmol/L) within 48 hours or by a 50% increase in sCr from baseline within 7 days, or a urine volume of less than 0.5 mL/kg/h for at least 6 hours
Urinary tract infection	Patient has:<br>a. at least one of the following signs of symptoms with no other recognised cause: fever (> 38 °C), urgency, frequency, dysuria, or suprapubic tenderness<br>b. and a positive urine culture, that is, $\geq 10^5$ microorganisms per ml of urine with no more than two species of microorganisms.<br>**Or**<br>Patient has:<br>a. at least two of the following with no other recognised cause: fever (> 38 °C), urgency, frequency, dysuria, or suprapubic tenderness,<br>b. and at least one of the following:<br>i. positive dipstick for leukocyte esterase and/or nitrate.<br>ii. pyuria urine specimen with ≥ 10 WBC/mL or ≥ 3 WBC/high-power field of unspun urine.<br>iii. organisms seen on Gram stain of unspun urine.<br>iv. at least two urine cultures with repeated isolation of the same uropathogen (gram-negative bacteria or S. saprophyticus) with $\geq 10^2$ colonies/mL urine in nonvoided specimens.

	v. $\leq 10^5$ colonies/mL of a single uropathogen (gram-negative bacteria or S. saprophyticus) in a patient being treated with effective antimicrobial agent for a urinary infection.<br>vi. physician diagnosis of a urinary tract infection.<br>vii. physician institutes appropriate therapy for a urinary infection
Surgical site infection	All 3 types of SSI (superficial incisional, deep incisional, organ or space) were considered as SSI in this study. Full definitions were:<br>Superficial incisional<br>Infection occurs within 30 days after the operation and involves only skin and subcutaneous tissue of the incision and at least one of the following:<br>a. purulent drainage with or without laboratory confirmation, from the superficial incision<br>b. organisms isolated from an aseptically obtained culture of fluid or tissue from the superficial incision.<br>c. at least one of the following signs or symptoms of infection: pain or tenderness, localised swelling, redness, or heat and superficial incision is deliberately opened by surgeon, unless incision is culture-negative.<br>d. diagnosis of superficial incisional SSI made by a surgeon or attending physician.<br><br>Deep incisional<br>Infection occurs within 30 days after the operation if no implant is left in place or within 90 days if implant is in place and the infection appears to be related to the operation and infection involves deep soft tissue (e.g. fascia, muscle) of the incision and at least one of the following:<br>a. purulent drainage from the deep incision but not from the organ/space component of the surgical site<br>b. a deep incision spontaneously dehisces or is deliberately opened by a surgeon when the patient has at least one of the following signs or symptoms: fever (> 38°C), localised pain or tenderness, unless incision is culture-negative.

	c. an abscess or other evidence of infection involving the deep incision is found on direct examination, during reoperation, or by histopathologic or radiologic examination.<br>d. diagnosis of deep incisional SSI made by a surgeon or attending physician.<br><br>Organ/space<br>Infection occurs within 30 days after the operation if no implant is left in place or within 90 days if implant is in place and the infection appears to be related to the operation and infection involves any part of the anatomy (e.g. organs and spaces) other than the incision that was opened or manipulated during an operation and at least one of the following:<br>a. purulent drainage from a drain that is placed through a stab wound into the organ/space.<br>b. organisms isolated from an aseptically obtained culture of fluid or tissue in the organ/space.<br>c. an abscess or other evidence of infection involving the organ/space that is found on direct examination, during reoperation, or by histopathologic or radiologic examination.<br>d. diagnosis of organ/space SSI made by a surgeon or attending physician.
Sepsis	SIRS criteria plus a definitive or presumed infectious foci.<br>SIRS was defined as two or more of the following:<br>• _Temperature < 36 °C or > 38 °C; or<br>• _Heart rate > 90 beats per minute; or<br>• _Respiratory rate) > 20 breaths per minute; or, PaCO2 < 32 mmHg; or<br>• _White blood cell count < 4000 cells/mm³ or > 12000 cells/mm³; or more than 10% immature neutrophils (band forms).

eTable 3 Sensitivity analysis for incorporating post-operative 30-day compilation as a confounding factor for long-term functional disability.

	Confounding with postoperative 30-day complication			Confounding without postoperative 30-day complication		
variable	OR	95%CI	*P* value	OR	95%CI	*P* value
Postoperative 6-month functional disability						
Time interval between COVID-19 diagnosis and surgery	1.00	0.99 ~ 1.00	<0.01	1.00	0.99~1.00	<0.01
Postoperative 12-month functional disability						
Time interval between COVID-19 diagnosis and surgery	0.99	0.98~1.00	0.01	0.99	0.99~1.00	0.01

SUPPLEMENTARY FIGURES

eFigure 1: Predicted Odds Ratios for 30-Day Postoperative Mortality.

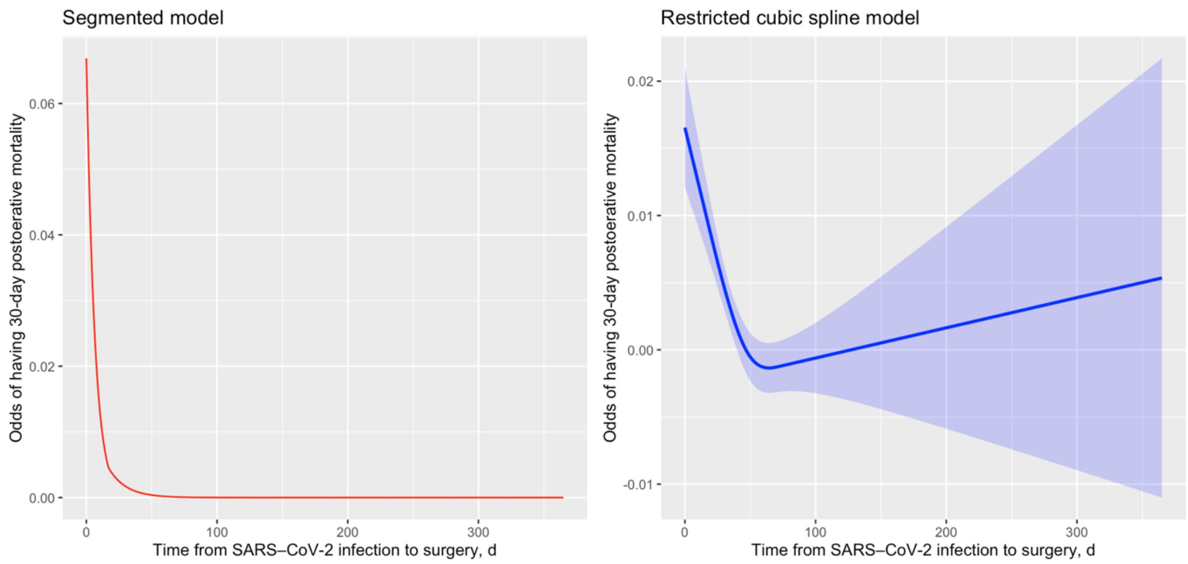

This figure illustrates the predicted odds ratios (ORs) for postoperative 30-day mortality, analyzed using a segmented model (left panel) and a restricted cubic spline model (right panel). The blue shaded area represents the 95% confidence interval (CI) for restricted cubic spline model.

eFigure 2: Predicted Odds Ratios for postoperative 6-month postoperative functional disability.

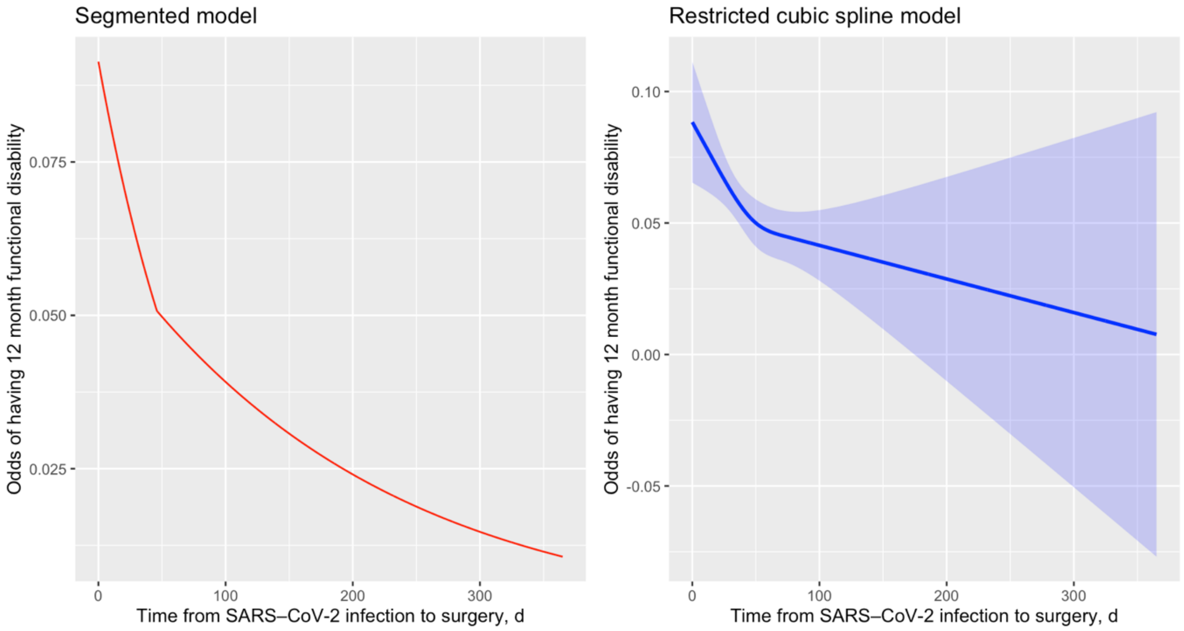

This figure illustrates the predicted odds ratios (ORs) for postoperative 6-month functional disability, analysed using a segmented model (left panel) and a restricted cubic spline model (right panel). The blue shaded area represents the 95% confidence interval (CI) for restricted cubic spline model.

eFigure 3: Predicted Odds Ratios for postoperative 12-month postoperative functional disability.

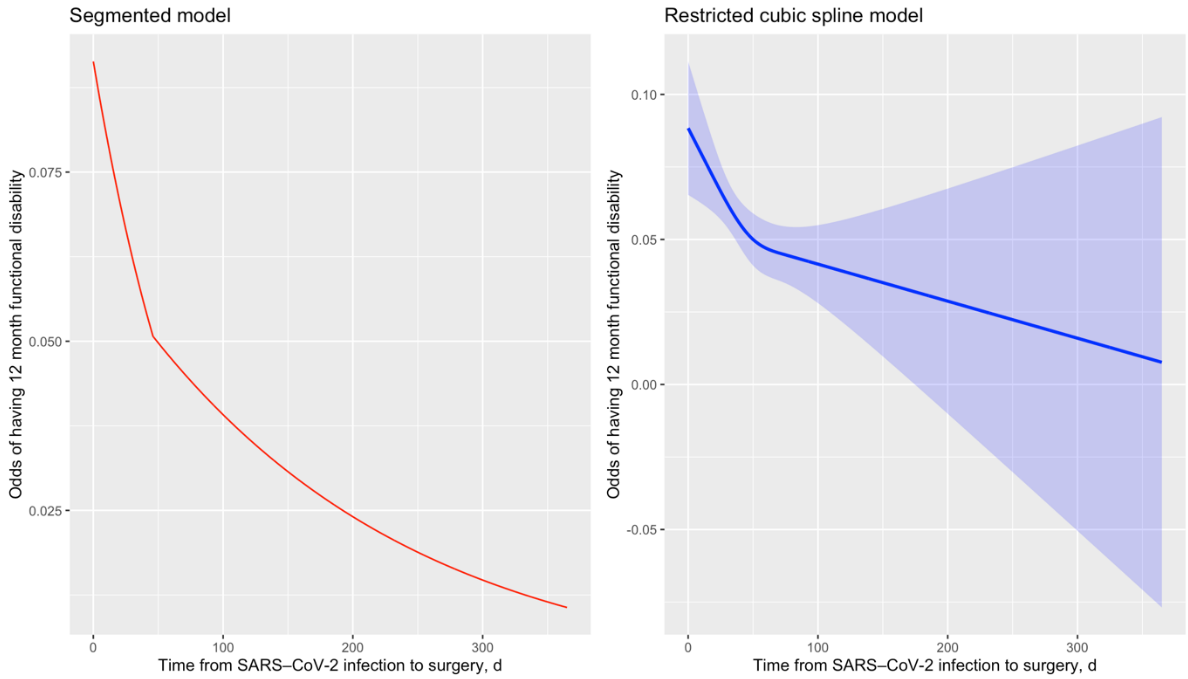

This figure illustrates the predicted odds ratios (ORs) for postoperative 12-month functional disability, analysed using a segmented model (left panel) and a restricted cubic spline model (right panel). The blue shaded area represents the 95% confidence interval (CI) for restricted cubic spline model.
